# A context-driven approach through stakeholder engagement to introduce a digital emergency obstetric and newborn care register into routine obstetric health care services in Bangladesh

**DOI:** 10.7189/jogh.14.04098

**Published:** 2024-05-10

**Authors:** Sabrina Jabeen, Rubaiya Matin Chandrima, Mehedi Hasan, Md Mahiur Rahman, Quazi Sadeq-ur Rahman, Tanvir Hossain AKM, Farhana Dewan, Azizul Alim, Nuzhat Nadia, Mustufa Mahmud, Moazzem Hossain Sarker, Jahurul Islam, Muhammad Shariful Islam, Sabina Ashrafee, Mohammad Sabbir Haider, Mohammod Jobayer Chisti, Md Ziaul Haque Sheikh, Mohammad Sanaullah Miah, Md Al-Mahmud, Shafiqul Ameen, Anisuddin Ahmed, Shams El Arifeen, Ahmed Ehsanur Rahman

**Affiliations:** 1 icddr,b (International Centre for Diarrhoeal Disease Research, Dhaka, Bangladesh; 2 Directorate General of Health Services, Ministry of Health and Family Welfare, Government of the People’s Republic of Bangladesh, Dhaka, Bangladesh; 3 Obstetrical and Gynaecological Society of Bangladesh (OGSB), Dhaka, Bangladesh

## Abstract

**Background:**

Emergency obstetric and newborn care (EmONC) in Bangladesh focusses on maternal health, whereby it addresses childbirth and postpartum complications to ensure women’s health and well-being. It was transitioned to a digital platform to overcome challenges with the paper-based EmONC register and we conducted implementation research to assess the outcome. Here we outline the stakeholder engagement process integral to the implementation research process.

**Methods:**

We adopted a four-step stakeholder engagement model based on the identification, sensitisation, involvement, and engagement of stakeholders. The approach was informed by previous experience, desk reviews, and expert consultations to ensure comprehensive engagement with stakeholders at multiple levels. Led by the Maternal Health Programme of the Government of Bangladesh, we involved high-power and high-interest stakeholders in developing a joint action plan for digitisation of the paper-based EmONC register. Finally, we demonstrated this digital EmONC register in real-life settings to stakeholders at different levels.

**Results:**

The successful demonstration process fostered government ownership and collaboration with multiple stakeholders, while laying the foundation for scalability and sustainability. Nevertheless, our experience highlighted that the stakeholder engagement process is context-driven, time-consuming, resource-intensive, iterative, and dynamic, and it requires involving stakeholders with varied expertise. Effective strategic planning, facilitation, and the allocation of sufficient time and resources are essential components for successful stakeholder engagement.

**Conclusions:**

Our experience demonstrates the potential of adopting the ‘identification, sensitisation, involvement, and engagement’ stakeholder engagement model. Success in implementing this model in diverse settings depends on leveraging knowledge gained during implementation, maintaining robust communication with stakeholders, and harnessing the patience and determination of the facilitating organisation.

Haemorrhage, hypertensive disorder, and severe infection causes more than half of the global maternal deaths. However, they can be avoided through ‘signal functions’ included in emergency obstetric and newborn care (EmONC), which is one of the most efficient strategies developed for combatting high maternal and neonatal mortality [1-3]. In Bangladesh, for instance, 44% of maternal deaths occur within the first 24 hours of delivery, where haemorrhage and eclampsia (which contribute to more than half of the deaths) can be easily prevented by the nine signal functions within comprehensive EmONC services [1,4]. Along with investments into such comprehensive care, an adept clinical data recording system is equally necessary for evidence-based decision-making centred on the recorded data [4-6], especially as the recording of patients’ health care data serves as the foundation for allocating resources, making informed decisions, and continually improving the quality of care [7-9].

Traditional paper-based registers have been used extensively for recording health care data. However, their inherent drawbacks led to a shift towards electronic or computerised and digital registers worldwide [10]. Similarly, emergency obstetrical health data in Bangladesh, including variables related to maternal and newborn care, are currently recorded in a paper-based register known as the EmONC register, which has been found to have the limitations of these conventional registers [11]. This register – a hardcover book containing tabulated data forms register – is currently used in the labour rooms of the public health facilities of Bangladesh [11]. Errors and data loss often occur due to multiple entry points and during duty shift changes, leading to overlapping records and challenges in merging information. The register is also not practical for real-time data monitoring and long-term preservation, making the data insecure and less reliable [12].

To address these existing challenges, the digital EmONC register was created as a replacement for the traditional paper-based system. We conducted implementation research to assess the implementation outcome of this digital system by using World Health Organization (WHO) implementation research variables [13]. The implementation of the digital EmONC register in Bangladesh also aimed to address critical challenges in data recording within maternal health services, replacing the fragmented paper-based systems with a comprehensive, reliable digital solution. This project was led by the Maternal Health Programme of the Directorate General of Health Services of Bangladesh, with technical assistance from icddr,b (formerly known as the International Centre for Diarrhoeal Disease Research, Bangladesh) and funding from the United States Agency for International Development (USAID) as part of the Research for Decision Makers activity [14,15]. Two district hospitals and two sub-district hospitals were selected for this implementation research, with the objective to integrate the digital EmONC register as the routine data recording system intended to replace the existing one.

A vital aspect of making research findings useful in local health systems is engaging a diverse group of stakeholders. Their involvement, in turn, secures support from health research funding organisations and aids in the effective implementation of research findings, while also generating impact [16,17]. Along with assessing the implementation outcome of the digital EmONC register, we also wanted to explore the integrated stakeholder engagement process and its key determinants, as they could influence the success and sustainability in transitioning from a paper-based to a digital EmONC register for maternal health services in Bangladesh. We could find no previous examples of the digitisation of an EmONC register through extensive stakeholder engagement in the public health care facilities. Based on our previous study, we applied the four-step stakeholder engagement model based on the identification, sensitisation, involvement, and engagement (ISIE) of stakeholders [18]. Specifically, by including key stakeholders in the design, development, implementation, and decision-making phases, we aimed to foster context-specific solutions, a participatory approach, government leadership, national ownership, scalability, and sustainability of the digital EmONC register.

## METHODS

### Approach: Adaptation of a conceptual framework for stakeholder engagement

**Figure 1:**
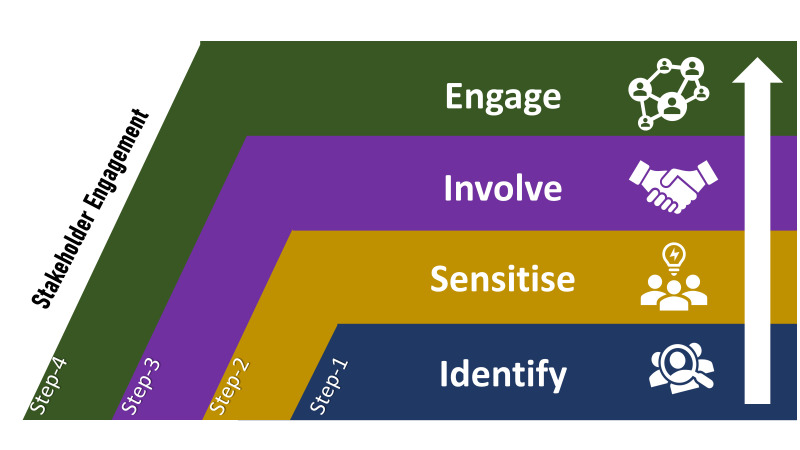
Conceptual framework adapted to guide the stakeholder engagement process in assessing the feasibility of a digital EmONC register in Bangladesh.

Stakeholders are individuals, entities, or local groups who possess the capacity to exert an impact on or be impacted by the progress and results of a project, research endeavour, or policy [19]. Existing literature focussing on engaging stakeholders in the field of health research highlights a variety of methods and strategies, largely originating from considerations of priorities, available opportunities, and specific contextual factors [16,20-22]. Using evidence from the literature, consultations with policymakers and experts, and practical experience gained from implementing development projects in the specific context of Bangladesh, we employed the ISIE stakeholder engagement model (**Figure 1**) [18] as a guide for the various activities related to stakeholder engagement in our study. It comprises four steps:

- Step 1: Identify and prioritise the stakeholders who will be further engaged and involved in the process;
- Step 2: Sensitise the majority of stakeholders to the research context, its rationale, significance, and the research process itself in order to build their interest, confidence, and trust in research;
- Step 3: Actively involve key stakeholders in the planning phase to ensure consensus on the implementation process and to solidify commitments from stakeholders regarding shared responsibilities;
- Step 4: Engage stakeholders continually at every stage, including the design, development, implementation, and evaluation of the research to ensure their ongoing participation and input throughout the project [18]

### Process steps taken to implement stakeholder engagement-related activities

#### Phase 1: Identifying stakeholders for sensitisation, involvement, and engagement

The Maternal Health Programme of the Directorate General of Health Services of Bangladesh is responsible for maintaining the EmONC register in public health facilities. To identify national and district-level stakeholders associated with the implementation of EmONC in Bangladesh, we extensively reviewed EmONC service development and implementation-related documents. We also purposefully selected seven key informants due to their involvement in maternal health services and interviewed them to identify the potential stakeholders based on their opinion. Following the saturation of data, we identified 11 national-level and 12 district-level stakeholder organisations. These included various government health programmes; professional bodies; United Nations (UN) agencies; local, national, and international organisations; and non-governmental organisations dedicated to delivering maternal health services in the Kushtia and Dinajpur district.

Our primary goal was to persuade policymakers and health care providers on the importance of transitioning from paper-based EmONC registers to digital records. To maximise limited resources, we prioritised a select group of stakeholder organisations that aligned with the project's goals. For this purpose, we derived a preliminary list of stakeholder organisations from the desk review and key informant interviews (Section ST1 and ST2 in the **Online Supplementary Document**). We then organised a consultative workshop with the Government of Bangladesh, in which participants (including the programme managers of the Maternal Health Programme and the National Newborn Health and IMCI Programme (NNHP & IMCI Programme) and their deputy programme managers) used a power-interest matrix to prioritise stakeholder organisations. Participants ranked these organisations on a 10-point Likert scale for their influence/power and interest in EmONC service implementation in Bangladesh [23].

**Figure 2:**
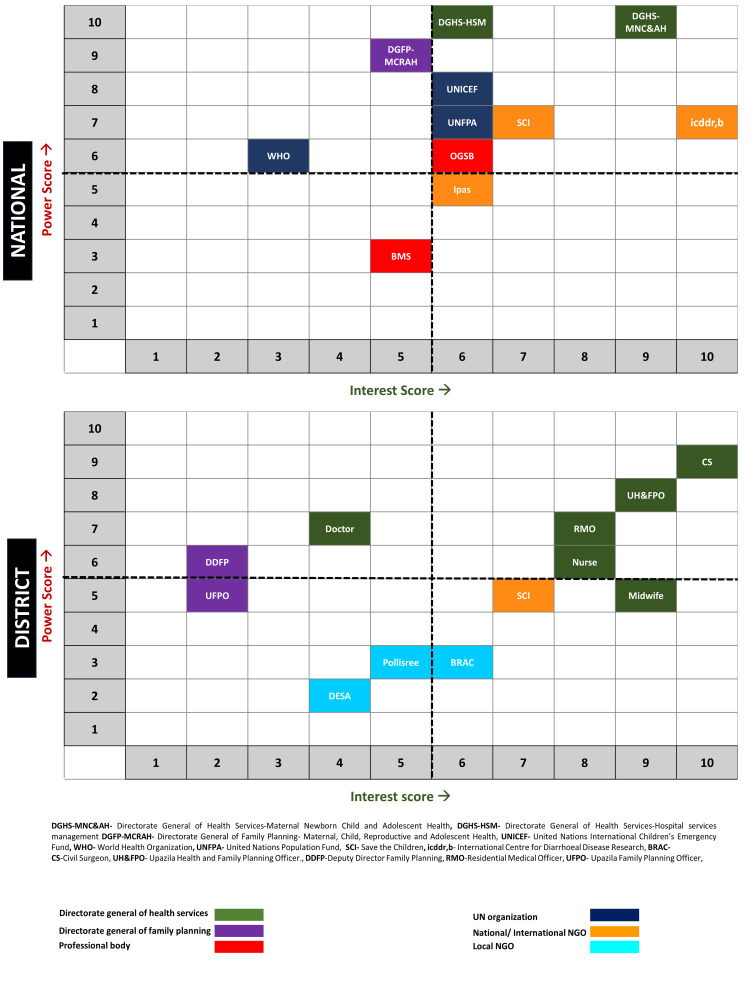
Power-interest mapping exercise of national and district level organisations related to EmONC service delivery in Bangladesh.

We then categorised stakeholder organisations with an average interest score of ≥6 as having high interest and those with an average power score of ≥6 as having high power. We then selected stakeholders which belonged to both the high interest and the high power group (i.e. high interest/high power) for further involvement and engagement stages (Section ST3 in the **Online Supplementary Document**). These stakeholders showed their interest in the project and contributed centrally to the data matrix development while also sharing their experience on working in the digital platform, which helped the digital application development team in developing the initial prototype. The central-level stakeholders also developed the implementation and evaluation plan for the digital application at the facility level. The peripheral level stakeholders, meanwhile, participated actively in the macro and micro plan development for the implementation of the digital EmONC register and executed it at the facility level (**Figure 2**).

#### Phase 2: Sensitising the stakeholders regarding the approach

First, based on the four categories created at the identification stage, we conducted a sensitisation workshop at the central level in which we engaged national-level stakeholders or policymakers. We selected our participants based on a previous sensitisation workshop chaired by the Line Director of Maternal, Neonatal, Child & Adolescent Health programme at the Directorate General of Health Services, which also involved national-level stakeholders or policymakers. In our workshop, the discussion focussed on the insufficient and inaccurate data on maternity and newborn health care services resulting from inefficiency of paper-based registers and its impact on the persistent high maternal mortality rates, highlighting the significance of shifting to digital register in alignment of the objectives of the proposed implementation research [13].

The stakeholders decided to digitise the existing paper-based EmONC register and implement it in four health care facilities in the Kushtia and Dinajpur districts to assess its feasibility. Consequently, two local-level sensitisation workshops were held in both districts, preceded by pre-sensitisation visits. In the workshops, the facility health managers (referred to locally as civil surgeons) from both districts; superintendents from both district hospitals, sub-district or *upazila* level health managers (known locally as *upazila* health and family planning officers); and district and sub-district (*upazila*) level stakeholder organisations were also invited to inform them about the project’s objective. We also invited national-level stakeholders (including managers from the Maternal Health Programme and the NNHP & IMCI Programme at the Directorate General of Health Services) and health care providers working in labour wards and obstetric operation theatres (including doctors, nurses, midwives, and statisticians in the selected implementing health facilities). In total, 73 participants attended both district sensitisation workshops, with 31 participants in the Kushtia and 42 participants in the Dinajpur district.

#### Phase 3: Involving the stakeholders in collaborative planning and role-sharing

We organised several consultative workshops at national, district, and sub-district levels among high power/high interest stakeholder organisations. During the national-level consultative workshops, the stakeholders collaborated to design the application and formulate the project implementation strategy for introducing a digital application-based EmONC register in health care facilities. It was collectively decided that icddr,b would serve as the focal point for application development and project implementation, while the other stakeholders would provide technical assistance and support.

At this stage, two local-level consultative meetings were held for each implementing facility, where the local facility managers and health care providers shared their insights on how they envisioned the project being implemented in their facilities. Then, a district-level action plan was developed which outlined the roles of district-level stakeholders and key actions, including training on the digital EmONC application; transitioning from paper-based registers; the introduction of tablet computer based digital registers in areas of the facilities providing delivery-related services; understanding the monthly reporting forms generated by the digital application; support for supervision visits; and plans for monitoring and evaluation. The sub-district level adopted a similar action plan, with facility managers and health care providers concurring on the project action plan and the distribution of responsibilities. A series of preparatory and planning meetings took place at the district and sub-district levels, resulting in the creation of facility-specific implementation plans including timelines.

#### Phase 4: Engaging stakeholders in design, development, and implementation

Development of the digital EmONC register application: During this phase, the Maternal Health Programme at the Directorate General of Health Services and icddr,b worked in close collaboration with the high power/high interest stakeholders. They focussed on designing and developing the digital EmONC register application, as well as implementing the project in the selected health care facilities.

**Figure 3:**
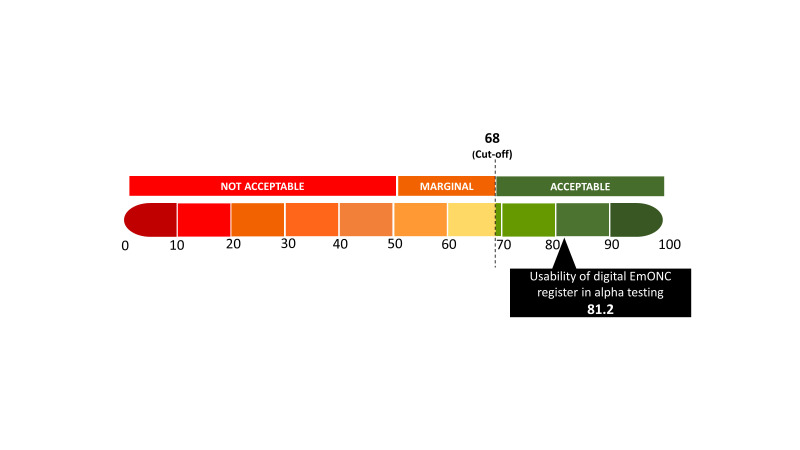
Usability of the alpha version of the digital EmONC register using SUS score (n = 12).

Stakeholders were actively engaged at every stage of the digital register development process. The research team at icddr,b collaborated with the in-house programme team involved in data management support. Following several meetings, this collaboration led to the development of the digital EmONC register application with a user manual, and an embedded resources library (Section ST4 in the **Online Supplementary Document**), and other features (**Figure 3**). Every stage of application development was presented to the Government and rest of the stakeholders for their opinion and agreement.

The development process had two main steps. First, the prototype formation and design (user experience/user interface) were finalised. During this stage, the research team at icddr,b engaged in an extensive process of mapping variables and developing the matrix for the digital EmONC register. They simultaneously conducted in-depth interviews with health care providers working in maternal health care services at the Dhaka Medical College and Hospital (a tertiary-level hospital) to gather their insights on the EmONC application’s user interface design [24]. Given that the digital EmONC register would serve as a service register, both the research and programme teams were committed to ensuring its user-friendliness and convenience.

**Figure 4:**
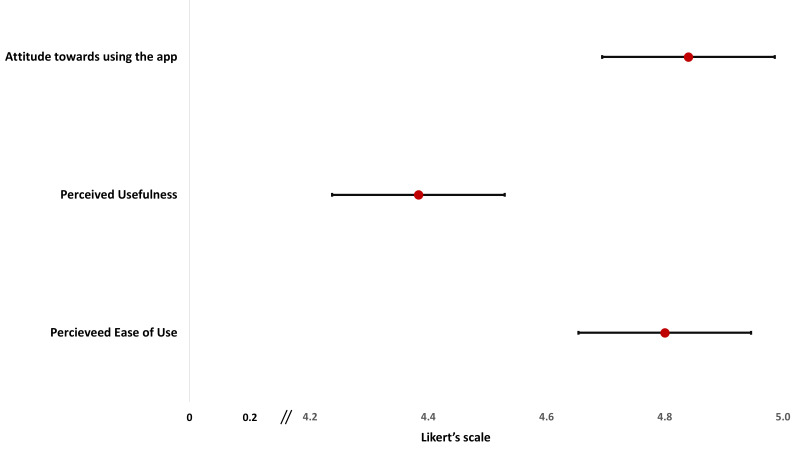
Acceptability of the alpha version of the digital EmONC register using the TAM score (n = 12).

The second step included the development and testing of the digital EmONC application. Following the feedback from stakeholders, an initial version of the application was developed. The research and programme teams conducted multiple rounds of quality assurance testing for the application, leading to the creation of the alpha version after necessary adjustments. Then, research physicians from the icddr,b assessed the usability and acceptability of the alpha version and scored it by using System Usability Scale score (**Figure 3**) and Technology Acceptance Model score (**Figure 4**), resulting in a more updated version after revisions [25-27]. During a consultative meeting, stakeholders recommended pre-testing this alpha version in a practical health care setting. Consequently, the application underwent pre-testing with health care providers at the Mohammadpur Fertility Services and Training Center. After responding to their feedback, the application was prepared for final approval by the Maternal Health Programme at the Directorate General of Health Services and other stakeholders.

A digital EmONC register webpage was also developed simultaneously. It was designed to be accessed by the local health care facility managers, statisticians at the health facilities, the Maternal Health Programme, and the icddr,b team.

**Figure 5:**
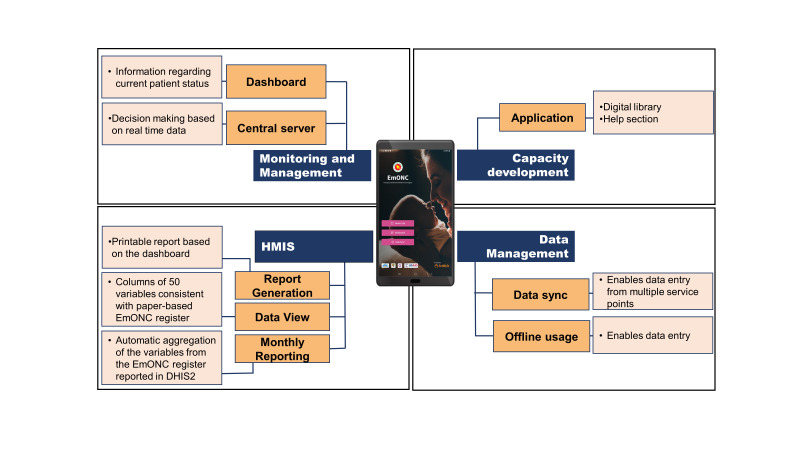
Features of the digital EmONC register.

Recognising the transition from a paper-based register to a digital platform, stakeholders recommended the creation of a user manual to assist health care providers in using the application. Consequently, user manuals were developed in both English and Bangla, incorporating the feedback from the stakeholders to guide the health care providers. The Bangla version of the manual was distributed to health care providers during training sessions, while the English version was provided to facility managers and policymakers (**Figure 5**).

The endorsement meeting for the digital EmONC register application took place at a national level, chaired by the Line Director of Maternal, Neonatal, Child, and Adolescent Health and was attended by the participants representing high-power/high-interest stakeholder organisations. During the meeting, the digital EmONC register received approval from the Line Director, and a unanimous agreement was reached on proceeding with the field implementation of the project.

Training of healthcare providers on the use of the digital EmONC register application: We also organised local-level training to train the health care providers working in the labour rooms and obstetrics operation theatres at the implementing health facilities (including nurses, midwives, and doctors) on how to use the digital EmONC register. These training sessions were held in the district-level health facilities and were attended by participants from both the district and the selected sub-district level (*upazila*) of the Kushtia and Dinajpur districts. The training package included the user manual, android tablet computers, and a list clarifying the roles of different service providers. The two-day training was structured to include theoretical and practical demonstrations of the application's functions. Fifty-four participants were trained in the Kushtia and 47 in Dinajpur district in six batches. The training was jointly conducted by the icddr,b research and the data management support team. The trainees’ feedback on the application was promptly addressed by the programme team.

Introduction of the digital EmONC register in the implementing health facilities: The digital EmONC register was introduced at the four implementing facilities following a collaborative effort among the icddr,b field team, facility managers, and health care providers. Each facility manager inaugurated this implementation, while the paper-based EmONC registers were discontinued on the same day. Healthcare providers began entering data into the EmONC application in the presence of the icddr,b team. The storage backup for this data entry was managed by the icddr,b central server team within the data management support section. The implementation of this project lasted three months, during which the icddr,b field team visited the health facilities daily in the first two weeks for active facilitation. Initially, health care providers required regular assistance from the icddr,b team for day-to-day technical issues management, but they gradually became proficient and independent.

Involving national and local level stakeholders in the implementation research: The implementation research protocol was developed using a co-design and co-creation approach involving the Maternal Health Programme, the NNH & IMCI Programme, and other stakeholder organisations representing the high power/high-interest groups, with icddr,b taking the lead. Stakeholders played a vital role in prioritising research questions and contributed to the research design, data collection techniques, and development of data collection instruments. Representatives from four stakeholder organisations, including the Maternal Health Programme, were integrated as co-investigators and scientific contributors, enhancing the comprehensiveness and relevance of the research protocol through a diverse set of inputs.

We held advisory meetings with local stakeholders at both the district and sub-district levels, at which we briefed them about the research questions and study design. A macro plan was developed in collaboration with facility managers to introduce the digital EmONC register and conduct the assessments outlined in the research protocol. Performance appraisal meetings were held in the facilities to acknowledge their contributions and celebrate their dedication to the project.

Lastly, we designed the project with multiple dissemination programmes which aimed to share the findings of the pilot implementation research with a special focus on the success of stakeholder engagement by using the ISIE model. Therefore, we organised both national and local-level programmes and invited stakeholders from all four groups. This approach ensured that the research findings reached and engaged a wide spectrum of stakeholders at various levels of interest and influence.

### Resources required for stakeholder engagement

**Figure 6:**
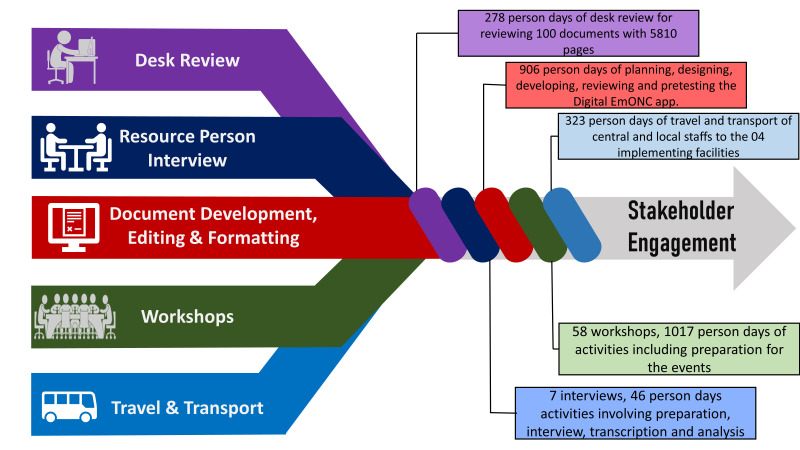
Resources required and invested for stakeholder engagement activities.

**Figure 6** shows the required resources for undertaking previously mentioned stakeholder engagement activities.

#### Desk review

In total, 278 person-days were dedicated to reviewing 90 documents with around 5810 pages to assess the feasibility and potential challenges, identify the stakeholders, and build on existing knowledge by medical doctors with basic obstetrics training.

#### Resource person interview

We conducted seven interviews with policymakers and lead obstetricians to identify and priorities the relevant stakeholders. This included obstetrics and gynaecology experts who helped to prioritise the identified stakeholder organisations. Besides the seven hours required for interviews, another 26 person-hours were dedicated for transcription and analysis.

#### Digital EmONC application and documents development

The application development process required 906 person-days of exhaustive efforts to plan, design, develop, review and pretest the digital EmONC app.

#### Workshops

A total of 1017 person-days of involvement were required to sensitise (step 2), involve (step 3), and engage (step 4) the stakeholders. Centrally, we arranged 25 workshops requiring approximately 510 person-days of active participation of maternal health experts, as well as 33 meetings requiring 117 person-days of involvement. Icddr,b invested 53 person-days for planning and organising these events.

#### Travel and transport

A total of 323 person-days of extensive travel and transport were required to sensitise (step 2), involve (step 3), and engage (step 4) the stakeholders. The central staff required 132 days of travel and local staff required 191 days of travel to the four implementing facilities.

### Timeline

**Figure 7:**
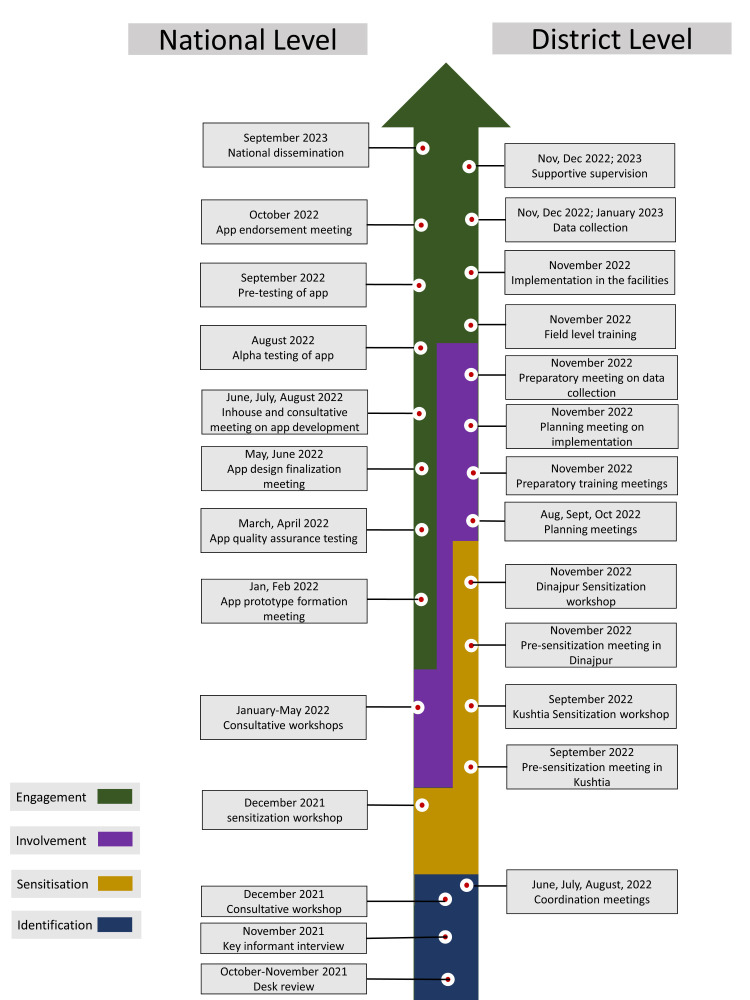
Milestones and timeline for stakeholder engagement activities.

**Figure 7** gives us the timeline for the main activities related to stakeholder engagement.

#### Phase 1: Identification

Both national and local stakeholders played a role during this stage. The national-level engagement started in October 2021 and continued until December 2021. Meanwhile, coordination meetings at the local level took place from June to August 2022 and helped with identifying local stakeholders.

#### Step 2: Sensitisation

We held a sensitisation workshop nationally in December 2021 and scheduled two local workshops, one in the Kushtia district in September 2022 and the other in the Dinajpur district in November 2022. We conducted pre-sensitisation meetings in the respective districts and sub-districts between September and November 2022 before the local sensitisation workshops.

#### Step 3: Involvement

The involvement stage at the national level lasted from January to May 2022. The timeline at the local level, meanwhile, was more fragmented. The stage started in August 2022 and concluded in November 2022, encompassing planning and preparatory meetings for the implementation of the digital EmONC register.

#### Step 4: Engagement

The process of engaging national-level stakeholders began in January 2022 and extended through to October 2022, culminating in the national endorsement of the project. We disseminated our findings in September 2023 while involving all relevant stakeholders. At the local level, the process began with training in November 2022 and wrapped up in mid-January 2023.

## RESULTS

### Impact of the stakeholder engagement process

#### Revolutionising obstetric services through stakeholder-driven digitisation

The Maternal Health Program, the NNH & IMCI Programme, and various other stakeholders recognised the limitations of the paper-based EmONC register and acknowledged the need for digitisation to create a more dependable data recording system. With this understanding, the government decided to introduce a digital EmONC register for routine obstetric services. To achieve scalability, a pilot initiative was launched, involving the training of 101 health care providers and the implementation of the digital EmONC register in selected health care facilities. In this process, the facilities replaced the traditional paper-based register with the digital EmONC register.

#### Immediate impact and scalability

The implementation of the digital EmONC register initially began in one of the 13 sub-district level health care facilities within the Dinajpur district and has since proven successful. This digital application has gained positive feedback from health care providers and facility managers, primarily due to its capacity to reduce workloads, streamline daily tasks, and enhance the reliability of delivery-related data collected from health care facilities. The civil surgeon of the Dinajpur district has expressed a strong interest in expanding the programme to the rest of all sub-district level health care facilities. In response to this request, the icddr,b team devised a strategy, collaborated closely with facility managers, and effectively extended the application to all sub-districts (*upazila*) level facilities across the Dinajpur district after providing training to 106 health care providers in 11 sub-districts.

#### Integrating to long-term sustainability by aligning with sector programmes

Given the success of the feasibility study for the digital EmONC register, the Government of Bangladesh decided to integrate the expansion of the digital EmONC register into the upcoming fifth Five-year Sector Programme of Bangladesh. Considering this development, the Maternal Health Programme has reached out to the management information system to secure a budget allocation for the acquisition of tablet computers in health care facilities. Here, the Maternal Health Programme will play a coordinating role in facilitating the training programme in collaboration with the management information system.

## DISCUSSION

**Figure 8:**
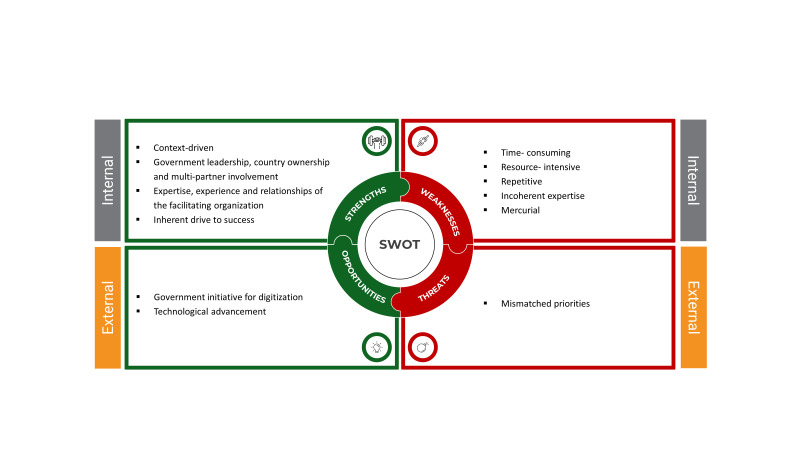
SWOT analysis of the stakeholder engagement process.

Using the strength, weakness, opportunity, and strength (SWOT) analysis approach, we have gathered valuable insights and findings from our stakeholder engagement efforts (**Figure 8**).

### Strengths

#### Context-driven

A study aimed at involving stakeholders in the implementation process should be flexible in adjusting its framework according to its specific goals and the surrounding circumstances. In our implementation research study, our main objective was to transition from a paper-based register to a digital platform that already existed. Considering the challenges associated with the paper-based system and how we intended to address them in the digital platform, we adopted a four-step model for engaging stakeholders. We focussed our efforts on prioritising high power/high interest organisations among the stakeholders. This model was our guiding principle throughout the main stages and in all related activities. Prioritising these stakeholders allowed us to make the most efficient use of our limited resources. A recent study found that adapting the ISIE framework to meet specific contextual challenges is a key aspect of stakeholder engagement strategies [18,28]. Our implementation research study similarly aimed to transition from a paper-based register to an existing digital platform, beginning with recognising the need to tailor our approach to the prevailing circumstances.

#### Government leadership, country ownership, and multi-partner involvement

The Maternal Health Programme of the Government of Bangladesh showcased its leadership capabilities by taking charge of workshops, formulating strategic implementation plans, and uniting various partners to establish a national consensus on transitioning from the paper-based EmONC register to a digital system. This approach, aligned with the WHO National eHealth Strategy Toolkit, emphasised the need for national-level planning, stakeholder engagement, and ongoing monitoring and evaluation of implementation processes, garnering support from professional bodies, prominent non-governmental organisations, and development partners actively involved in maternal health systems related activities in Bangladesh [29]. They contributed their technical expertise and resources to further the implementation of this project, with the shared objective of strengthening health system initiatives. Similarly, a study on sustainable health care transitions underscored the significance of multi-stakeholder involvement and collaborative foresight for long-term transition strategies [30].

#### Expertise, experience, and relationships of the facilitating organisation

icddr,b is an international research institution located in Bangladesh with over 60 years of experience in collaborating with the Government, resulting in significant improvements in our country's maternal health system. Their track record of successful endeavours and extensive experience greatly helped identify, raise the awareness of, and engage other high power/high interest stakeholder organisations. Moreover, icddr,b also boasts a data management support system capable of developing and overseeing the digital platform's operation nationwide. Additionally, their adept communication and organisational skills played a pivotal role in efficiently facilitating the engagement of stakeholders. Therefore, it is crucial to consider the positioning and capabilities of implementing organisations when selecting an approach for stakeholder engagement. Studies conducted in different settings emphasised the importance of considering the positioning and capabilities of implementing organisations when selecting an approach for stakeholder engagement in adaptation interventions [18,31]. This is particularly relevant in developing nations, where low institutional capacity can constrain project success.

#### Inherent drive to success

The field team of icddr,b maintained constant communication with facility managers, keeping them informed about upcoming activities. The significant impact of these activities contributed to individual facility-level achievements. This indirect influence played a crucial role in convincing facility managers about the benefits of adopting the digital EmONC register. Therefore, the eagerness shown by some facility managers to embrace this new technology emerged as a major driving force behind the successful project implementation, ultimately leading to its expansion throughout the Dinajpur district. Individual stakeholder's personal motivation for change can be a potent asset in the implementation process, and achieving this often involves one-on-one sensitisation of key personnel in various organisations. Similarly, Postema et al. [32] emphasised that an individual stakeholder's motivation for change can significantly impact the implementation process of innovations.

### Weakness

#### Time-consuming

The process of implementing stakeholder engagement activities and accomplishing the objective of integrating the digital EmONC register into the fifth Five-year Sector Programme took approximately one and a half years. We conducted several workshops, both centrally and at the field level, based on the availability of all high power/high interest stakeholders. This proved challenging due to their busy schedules. Additionally, extensive updates were needed to incorporate feedback from the previous meetings into the digital application. This necessitated several days or weeks of work for the application development team before these workshops could be organised. Different studies have shown that effective stakeholder engagement is time-consuming, demanding patience and unwavering commitment from the facilitating organisation [18,33].

#### Resource-intensive

The initial phase of the project involved an extensive document review to identify stakeholders, as well as the design and development of the digital EmONC register application based on a complex algorithm. This undertaking demanded many person-hours and dedication from the research and application development team members. Furthermore, organising workshops at the national, district, and sub-district levels required the participation of maternal health experts, facility managers, service providers, the application development team and the research team, likewise necessitating many person-days. As the project progressed, the placement of tablet computers to suit facility requirements and ensure their demanded security incurred additional costs, as well as extensive travel for project staff. Given that stakeholder engagement is a resource-intensive process, it is imperative to have a well-thought-out plan and a dedicated budget allocation for stakeholder engagement right from the onset of any project.

#### Repetitive

Examining the variable matrix, making updates based on various patient condition scenarios and outcomes, and then integrating these changes into the digital application through adjustments to the algorithm was a notably extensive and repetitive process. The application development team aimed to consider the feedback from stakeholders at the national, district, and sub-district levels before the official launch. As a result, the variable matrix underwent 26 rounds of revision. Simultaneously, the user manual for the developing application underwent 12 revisions to synchronise with the application itself. Hence, the facilitating organisation must demonstrate dedication, meticulous attention to detail, and sometimes patience for repetitive work to manage the stakeholder engagement process effectively [18,33,34].

#### Incoherent expertise

Transitioning from a paper-based register used in the labour room for recording delivery and newborn-related information to a digital platform and evaluating its feasibility necessitated a team of experts comprising maternal health specialists, implementation research experts, and digital programming experts. While the maternal health experts and the research team had prior experience working together, integrating the programming team proved challenging initially due to differences in programming languages and the need to convey maternal health-related concepts and terminology to the programming team. In this context, the research team played a crucial role as a bridge between the other teams. Previous studies also faced challenges of bringing individuals with different expertise to work on a single platform by creating their own dynamics [35,36]. Seeing these challenges, the facilitating organisation must show patience and persistence in fostering connections among stakeholders from various domains.

#### Mercurial

The power-interest matrix of stakeholders is not fixed and can undergo changes at any point. For instance, within the engagement process, a stakeholder initially characterised as having high power/high interest unexpectedly shifted to a high power/low interest role and began disengaging from decision-making. Conversely, another stakeholder with high power/high interest introduced additional project requirements during the engagement, leading the project team to adjust the training duration and method. Furthermore, leadership changes can occur during stakeholder engagement; for example, the district-level facility manager was transferred during the implementation, demanding to the need for extra time and effort to acquaint the new stakeholders with the project and secure their active support for introducing the digital register. During the implementation period, the role shifting due to the change of power dynamics was evident in our previous study with heavily embedded stakeholder engagement [18]. However, stakeholder engagement is an adaptable process, demanding flexibility in revising, updating, and amending the plan according to prevailing contexts and circumstances.

### Opportunity

#### Government initiative for digitisation

The Government of Bangladesh is committed to the digitalisation of the health care sector [37]. In line with this vision, the Ministry of Health and Family Welfare has been actively implementing various measures to leverage digital solutions to enhance the accessibility, quality, and affordability of health care services. Consequently, this presented a significant opportunity to highlight the importance of transitioning from a paper-based register to a digital one in labour rooms to stakeholders and rally their support for this transformation. Katherin et al. [38] acknowledged the involvement of the implementing country’s government in safeguarding the significant emphasis on user-interface, favouring iterative approaches over the traditional waterfall methods, and establishing a design principle to ensure consistency and enhance user experience.

#### Technological advancement

In the age of digital solutions, many people are using Android-based mobile phones and are actively connected to social media. These factors played a crucial role in helping stakeholders recognise the benefits of a digital solution compared to a paper-based one, particularly its capacity to decrease workloads and deliver real-time data. Furthermore, during the training phase, health care providers were able to grasp and adapt to the application more quickly than anticipated. Even after implementation, trainee health care providers found this new technology intriguing and voluntarily invested their time in learning how to use it effectively.

### Threats

#### Mismatched priorities

The development of the digital register under the direct supervision of central-level stakeholders posed certain challenges when it came to its implementation at peripheral-level facilities. This included, for example, ensuring smooth integration between these two layers and gaining acceptance. To address this, the programme team worked on persuading central-level stakeholders to visit peripheral facilities to build trust and confidence in the programme. However, this proved to be extremely challenging due to the difficulty of finding suitable timing and alignment with their busy schedules. For instance, there were three instances of last-minute cancellations of peripheral-level sensitisation programmes by central-level stakeholders, which created tension between the two layers. The facilitating organisations had to take on an active and strategic role in facilitating collaboration and bringing these two layers together on a common platform. Referring to the stakeholder engagement activities conducted in an implementation research the Kushtia district of Bangladesh, such mismatches of priorities were evident in our previous study [18]. Nevertheless, it is important to recognise the possible impact of a new threat on the final results and schedule of the stakeholder engagement process. There should be room for adjusting the plan to implement corrective actions when needed [28].

### Limitations

In this study, we could not capture the long-term implications, challenges, or changes that occurred after the initial implementation phase due to the relatively short duration of the research. Likewise, communicating complex technical details from the health care domain to the programming team has introduced communication barriers; however, this was addressed through multiple in-house meetings and testing of the software by medical doctors. Lastly, we used the four-step ISIE model for stakeholder engagement, leaving a gap for other models that could have been tested.

## CONCLUSIONS

Our implementation research focussed on assessing the implementation outcome of the digital EmONC register played a crucial role in its integration into the ‘National Sector Plan,’ paving the way for scalability and sustainability. The success of integrating the digital register into the national health care system is not just contingent on technological feasibility, but is also heavily reliant on comprehensive stakeholder involvement. We followed a four-step ISIE stakeholder engagement model, which has again demonstrated its substantial value. Through our experience, we have learned that this model is highly context-specific, time-consuming, resource-intensive, iterative, and often unpredictable, as it requires involving stakeholders with diverse expertise, which necessitates strategic planning and effective facilitation. Using this ISIE stakeholder engagement model for country-wide scaling up of the digital EmONC register can serve as a globally replicable example for digitisation in the health sector.

## Additional material


Online Supplementary Document

## References

[1] Jabeen S Siddique AB Hossain AT Khan S Haider MM Tahsina T Haemorrhage-related maternal mortality in Bangladesh: Levels, trends, time of death, and care-seeking practices based on nationally representative population-based surveys. J Glob Health. 2023;13:07001. 10.7189/jogh.13.07001 37022713 PMC10080499

[2] Say L Chou D Gemmill A Tunçalp Ö Moller A-B Daniels J Global causes of maternal death: a WHO systematic analysis. Lancet Glob Health. 2014;2:e323-33. 10.1016/S2214-109X(14)70227-X 25103301

[3] World Health Organization. Monitoring emergency obstetric care, a handbook. Geneva: World Health Organization; 2009. Available: https://www.who.int/publications/i/item/978. Accessed: 22 October 2023.

[4] Hossain AT Siddique AB Jabeen S Khan S Haider MM Ameen S Maternal mortality in Bangladesh: Who, when, why, and where? A national survey-based analysis. J Glob Health. 2023;13:07002. . 10.7189/jogh.13.07002 37288544 PMC10248997

[5] Paxton A Maine D Freedman L Fry D Lobis S The evidence for emergency obstetric care. Int J Gynaecol Obstet. 2005;88:181-93. 10.1016/j.ijgo.2004.11.026 15694106

[6] Otolorin E Gomez P Currie S Thapa K Dao B Essential basic and emergency obstetric and newborn care: from education and training to service delivery and quality of care. Int J Gynaecol Obstet. 2015;130:S46-53. 10.1016/j.ijgo.2015.03.007 26115858

[7] Lorkowski J Pokorski M Medical records: A historical narrative. Biomedicines. 2022;10:2594. 10.3390/biomedicines10102594 36289856 PMC9599146

[8] Godbole M Agarwal A Clinical data driven decision support in healthcare informatics. Int J Eng Res Technol (Ahmedabad). 2020;13:107-16. 10.37624/IJERT/13.1.2020.107-116

[9] Subagja IK Amaliyah N Hiermy U Rahardjo BT Laxmi E Lydia KS Evaluation of Big Data analytics in medical science. Int J Eng Adv Technol. 2019;8:717-20. 10.35940/ijeat.F1132.0986S319

[10] Murphy C Keogh I The evolution of the medical record from paper to digital: an ENT perspective. J Laryngol Otol. 2023;137:246-8. 10.1017/S0022215122002018 36093953

[11] Khan MAH Cruz VO Azad AK Bangladesh’s digital health journey: reflections on a decade of quiet revolution. WHO South-East Asia J Public Health. 2019;8:71-6. 10.4103/2224-3151.264849 31441440

[12] Pourasghar F Malekafzali H Kazemi A Ellenius J Fors U What they fill in today, may not be useful tomorrow: lessons learned from studying Medical Records at the Women hospital in Tabriz, Iran. BMC Public Health. 2008;8:139. 10.1186/1471-2458-8-139 18439311 PMC2377263

[13] World Health Organization. Implementation Research in Health: A practical guide. Geneva: World Health Organization; 2013. Available: https://iris.who.int/bitstream/handle/10665/91758/9789241506212_eng.pdf?sequence=1. Accessed: 22 October 2023.

[14] icddr b. Main Page. 2024. Available: https://www.icddrb.org/. Accessed: 22 October 2023.

[15] United States Agency for International DevelopmentMain Page. 2024. Available: https://www.usaid.gov/. Accessed: 22 October 2023.

[16] Boaz A Hanney S Borst R O’Shea A Kok M How to engage stakeholders in research: design principles to support improvement. Health Res Policy Syst. 2018;16:60. 10.1186/s12961-018-0337-6 29996848 PMC6042393

[17] Huzzard T Achieving impact: Exploring the challenge of stakeholder engagement. Eur J Work Organ Psychol. 2021;30:379-89. 10.1080/1359432X.2020.1761875

[18] Rahman AE Jabeen S Fernandes G Banik G Islam J Ameen S Introducing pulse oximetry in routine IMCI services in Bangladesh: A context-driven approach to influence policy and programme through stakeholder engagement. J Glob Health. 2022;12:06001. 10.7189/jogh.12.06001 35441007 PMC8994831

[19] Deverka PA Lavallee DC Desai PJ Esmail LC Ramsey SD Veenstra DL Stakeholder participation in comparative effectiveness research: defining a framework for effective engagement. J Comp Eff Res. 2012;1:181-94. 10.2217/cer.12.7 22707880 PMC3371639

[20] Concannon TW Fuster M Saunders T Patel K Wong JB Leslie LK A systematic review of stakeholder engagement in comparative effectiveness and patient-centered outcomes research. J Gen Intern Med. 2014;29:1692-701. 10.1007/s11606-014-2878-x 24893581 PMC4242886

[21] Concannon TW Grant S Welch V Petkovic J Selby J Crowe S Practical guidance for involving stakeholders in health research. J Gen Intern Med. 2019;34:458-63. 10.1007/s11606-018-4738-6 30565151 PMC6420667

[22] Mallery C Ganachari D Smeeding J Fernandez J Lavalle D Siegel J Innovative Methods for Stakeholder Engagement: An Environmental Scan. Value Health. 2012;15:A14. 10.1016/j.jval.2012.03.082

[23] Jebb AT Ng V Tay L A review of key Likert scale development advances: 1995–2019. Front Psychol. 2021;12:637547. 10.3389/fpsyg.2021.637547 34017283 PMC8129175

[24] Bollini L Beautiful interfaces. From user experience to user interface design. The Design Journal. 2017;20:S89-101. 10.1080/14606925.2017.1352649

[25] Muñoz-Neira C López OL Riveros R Núñez-Huasaf J Flores P Slachevsky A The technology–activities of daily living questionnaire: a version with a technology-related subscale. Dement Geriatr Cogn Disord. 2012;33:361-71. 10.1159/000338606 22797087 PMC4722866

[26] Jamal A Tharkar S Alenazi H Julaidan BS Al Hindawi DA AlAkeel NS Usability analysis of a health sciences digital library by medical residents: cross-sectional survey. JMIR Form Res. 2021;5:e23293. 10.2196/23293 34184992 PMC8277327

[27] Kalayou MH Endehabtu BF Tilahun B The applicability of the modified technology acceptance model (TAM) on the sustainable adoption of eHealth systems in resource-limited settings. J Multidiscip Healthc. 2020;13:1827-37. 10.2147/JMDH.S284973 33299320 PMC7721313

[28] Brunetti F Matt DT Bonfanti A De Longhi A Pedrini G Orzes G Digital transformation challenges:strategies emerging from a multi-stakeholder approach. TQM J. 2020;32:697-724. 10.1108/TQM-12-2019-0309

[29] World Health Organization. National eHealth Strategy Toolkit. Geneva: World Health Organization; 2012. Available: https://www.who.int/publications/i/item/national-ehealth-strategy-toolkit. Accessed: 22 October 2023.

[30] Pereno A Eriksson D A multi-stakeholder perspective on sustainable healthcare: From 2030 onwards. Futures. 2020;122:102605. 10.1016/j.futures.2020.102605 32834076 PMC7375280

[31] Sherman M Ford JD Stakeholder engagement in adaptation interventions: an evaluation of projects in developing nations. Clim Policy. 2014;14:417-41. 10.1080/14693062.2014.859501

[32] Postema T Groen AJ Krabbendam KJJ A model to evaluate stakeholder dynamics during innovation implementation. Int J Innov Manage. 2012;16:1250025. 10.1142/S136391961200385X

[33] Mott Lacroix KE Megdal SB Explore, synthesize, and repeat: Unraveling complex water management issues through the stakeholder engagement wheel. Water. 2016;8:118. 10.3390/w8040118

[34] Zabell T Long KM Scott D Hope J McLoughlin I Enticott J Engaging Healthcare Staff and Stakeholders in Healthcare Simulation Modeling to Better Translate Research Into Health Impact: A Systematic Review. Front Health Serv. 2021;1:644831. 10.3389/frhs.2021.644831 36926474 PMC10012644

[35] Øvretveit J Scott T Rundall TG Shortell SM Brommels M Improving quality through effective implementation of information technology in healthcare. Int J Qual Health Care. 2007;19:259-66. 10.1093/intqhc/mzm031 17717038

[36] Souther E Implementation of the electronic medical record: the team approach. Comput Nurs. 2001;19:47-55. 11280148

[37] Alam MZ Hu W Uddin MA Digital transformation in healthcare services sector of Bangladesh: Current status, challenges and future direction. Journal on Innovation and Sustainability RISUS. 2020;11:30-8. 10.23925/2179-3565.2020v11i1p30-38

[38] Benjamin K Potts HW Digital transformation in government: Lessons for digital health? Digit Health. 2018;4:2055207618759168. 10.1177/2055207618759168 29942624 PMC6005404

