# Supplementary material for: A context-driven approach through stakeholder engagement to introduce a digital emergency obstetric and newborn care register into routine obstetric health care services in Bangladesh
Source: J Glob Health. 2024 May 10;14:04098. doi: 10.7189/jogh.14.04098 (PMC11079701; doi:10.7189/jogh.14.04098)
Supplement: Online Supplementary Document [file jogh-14-04098-s001.pdf]

Supplementary Material

Online Supplementary Document

Table ST1: Documents reviewed for developing digital EmONC register

SL	Name of Documents	Pages
1	WHO Recommendation for The Prevention and Treatment of Postpartum Haemorrhage (PPH)	48
2	Eclampsia and PPH Action Plan in Bangladesh	42
3	Guideline on Antenatal Care (ANC) (Bangladesh)	41
4	Maternal Health Standard Operating Procedure (SOP) Volume 1	226
5	Maternal Health Standard Operating Procedure (SOP) Volume 2	384
6	Bangladesh National Strategy for Maternal Health (English)	90
7	Bangladesh National Strategy for Maternal Health (Bangla)	90
8	Standard Clinical Management Protocols and Flowcharts on Emergency Obstetric and Neonatal Care 2019	132
9	Manual on Labour Room Management Protocol	161
10	Guideline on Intrapartum care (IPC) and Postnatal Care (PNC)	66
11	Monthly reporting form	1
12	Guidelines of Paper Based Emonc Register	4
13	National Institute of Population Research and Training (NIPORT), International Centre for Diarrhoeal Disease Research Bi, b),, MEASURE Evaluation. Bangladesh Maternal Mortality and Health Care Survey 2016 Final Report. Dhaka, Bangladesh, and Chapel Hill, NC, USA: NIPORT, icddr,b, and MEASURE Evaluation.; 2019.	357
14	MIS-Health, DGHS. Emergency Obstetric Care (EmONC) Performance Report 2011	20
15	Bailey P, Lobis S, Maine D, Fortney JA. Monitoring emergency obstetric care: a handbook: World Health Organization; 2009	164
16	Bodart TLRSC. Design and implementation of health information systems 2000	280
17	Asah FN, Kanjo C, Msendema MB, Addo H, Logo DDJJoHIiA. The Digitalization of Routine Data Management at the Point-Of-Care: The case of Ghana. 2020;7(2):18-28.	274
18	Biswas A. Shifting paradigm of maternal and perinatal death review system in Bangladesh: A real time approach to address sustainable developmental goal 3 by 2030 [version 1; peer review: 2 approved]. F1000Research. 2017;6(1120)	5
19	Khan M, Cruz V, Azad A. Bangladesh’s digital health journey: reflections on a decade of quiet revolution. WHO South-East Asia Journal of Public Health. 2019;8(2):71-6	5

20	Khalil-Ur-Rahmen YRFMSZTAA, Stergioulas KMAALK. Current Challenges of Digital Health Interventions in Pakistan:	91
21	Mixed Methods Analysis. JOURNAL OF MEDICAL INTERNET RESEARCH. 2020	5
22	Bangladesh Bureau of Statistics (BBS), Ministry of Planning, Government of the People's Republic of Bangladesh. District Statistics 2011 Kushtia. 2011	145
23	Bangladesh Bureau of Statistics (BBS), Ministry of Planning, Government of the People's Republic of Bangladesh. District Statistics 2011 Kurigram. 2011.	121
24	Press CU. alpha version 2021, https://dictionary.cambridge.org/dictionary/english/alpha-version.	2
25	Press CU. beta version 2021, https://dictionary.cambridge.org/dictionary/english/beta-version.	2
26	Bangor A, Kortum PT, Miller JTJIJoHCI. An empirical evaluation of the system usability scale. 2008;24(6):574-94.	20
27	Joo HJIJoAER. A study on understanding of UI and UX, and understanding of design according to user interface change. 2017;12(20):9931-5.	5
28	Kim S-J, Cho D-EJRoKCA. Technology trends for UX/UI of smart Contents. 2016;14(1):29-33.	2
29	Ruysen H, Rahman AE, Gordeev VS, Hossain T, Basnet O, Shirima K, et al. Electronic data collection for multi-country, hospital-based, clinical observation of maternal and newborn care: EN-BIRTH study experiences. BMC Pregnancy Childbirth. 2021;21(1):234.	15
30	Ng AW, Lo H, Chan A, editors. Measuring the Usability of Safety Signs: A use of system usability scale (SUS). proceedings of the International MultiConference of Engineers and Computer Scientists; 2011: Citeseer.	7
31	Brooke J. System Usability Scale (SUS): A quick and dirty usability scale. 1996.	8
32	. Bangor A, Kortum PT, Miller JT. An empirical evaluation of the system usability scale. Int J Hum Comput Interact. 2008;24(6):574-94.	22
33	Davis FD. A technology acceptance model for empirically testing new end-user information systems: Theory and results: Massachusetts Institute of Technology; 1985.	290
34	Davis FDJMq. Perceived usefulness, perceived ease of use, and user acceptance of information technology. 1989:319-40.	24
35	Gillenson ML, Sherrell DLJI, management. Enticing online consumers: an extended technology acceptance perspective. 2002;39(8):705-19.	15

36	Weiers RM. Introduction to Business Statistics2011.	892
37	Hossain P, Das Gupta R, YarZar P, Salieu Jalloh M, Tasnim N, Afrin A, et al. 'Feminization'of physician workforce in Bangladesh, underlying factors and implications for health system: Insights from a mixed-methods study. 209;14(1):e0210820.	21
38	Research NIoP, Training - NIPORT, Health Mo, Family Welfare, ICF. Bangladesh Demographic and Health Survey 2017-18. Dhaka, Bangladesh: NIPORT/ICF; 2020.	92
39	Research NIoP, Training - NIPORT, Health Mo, Family Welfare, ICF. Bangladesh Demographic and Health Survey 2011. Dhaka, Bangladesh: NIPORT/ICF; 2020.	458
40	Naeye RL, Burt LS, Wright DL, Blanc WA, Tatter DJP. Neonatal mortality, the male disadvantage. 1971;48(6):902-6.	2
41	Ahmed1, 2 S, Khan AKS, Noushad S. Early marriage; a root of current physiological and psychosocial health burdens. 2014.	5
42	. Mehra D, Sarkar A, Sreenath P, Behera J, Mehra SJBph. Effectiveness of a community based intervention to delay early marriage, early pregnancy and improve school retention among adolescents in India. 2018;18(1):1-13.	13
43	. Ahmed S, Sobhan F, Islam AJJotp. Neonatal morbidity and care-seeking behaviour in rural Bangladesh. 2001;47(2):98-105.	8
44	Chukuezi CJRjoss. Socio-cultural factors associated with maternal mortality in Nigeria. 2010;1(5):22-6.	6
45	Unicef I, UNICEF. Annual report 2012: Unicef; 2013.	50
46	Sumankuuro J, Mahama MY, Crockett J, Wang S, Young JJBp, childbirth. Narratives on why pregnant women delay seeking maternal health care during delivery and obstetric complications in rural Ghana. 2019;19(1):1-13.	13
47	UNICEF. Women's Health [cited 2017 19 October]. Available from: https://www.unicef.org/bangladesh/health_nutrition_407.htm.	1
48	36. Ahmed S, Sobhan F, Islam A. Neonatal Morbidity and Care-seeking Behaviour in Rural Bangladesh. Journal of Tropical Pediatrics. 2001;47(2):98-105.	8
49	Rosenstock S, Katz J, Mullany LC, Khatry SK, LeClerq SC, Darmstadt GL, et al. Sex differences in morbidity and care-seeking during the neonatal period in rural southern Nepal. 2015;33(1):1-10.	10
50	UNICEF. UNICEF Annual Report. 2012.	52
51	Roy SK, Jolly SP, Shafique S, Fuchs GJ, Mahmud Z, Chakraborty B, et al. Prevention of malnutrition among young children in rural Bangladesh by a food-health-care	9

	educational intervention: a randomized, controlled trial. Food and nutrition bulletin. 2007;28(4):375-83.	
52	Joshi S. Female household-headship in rural Bangladesh: incidence, determinants and impact on children's schooling. 2004.	50
53	Aktar S, Sachu MK, Ali ME. The impact of rewards on employee performance in commercial banks of Bangladesh: an empirical study. Journal of Business and Management. 2012;6(2):9-15.	8
54	UNICEF. The state of the world's children 2007: women and children: the double dividend of gender equality: Unicef; 2006.	160
55	Nigatu D, Gebremariam A, Abera M, Setegn T, Deribe K. Factors associated with women's autonomy regarding maternal and child health care utilization in Bale Zone: a community based cross-sectional study. BMC women's health. 2014;14(1):79.	9
56	Victora CG, Wagstaff A, Schellenberg JA, Gwatkin D, Claeson M, Habicht J-P. Applying an equity lens to child health and mortality: more of the same is not enough. The Lancet. 2003;362(9379):233-41.	9
57	Newham J, Alderdice F. If gender matters in maternity care, does it matter in maternity care research? : Taylor & Francis; 2017.	4
58	Nigatu D, Gebremariam A, Abera M, Setegn T, Deribe KJBwsh. Factors associated with women's autonomy regarding maternal and child health care utilization in Bale Zone: a community based cross-sectional study. 2014;14(1):1-9.	9
59	Betron ML, McClair TL, Currie S, Banerjee JJRh. Expanding the agenda for addressing mistreatment in maternity care: a mapping review and gender analysis. 2018;15(1):1-13.	13
60	Parveen S. Access of rural women to productive resources in Bangladesh: a pillar for promoting their empowerment. International Journal of Rural Studies. 2008;15(1).	2
61	Black RE, Allen LH, Bhutta ZA, Caulfield LE, De Onis M, Ezzati M, et al. Maternal and child undernutrition: global and regional exposures and health consequences. The lancet. 2008;371(9608):243-60.	18
62	Rosenstock S, Katz J, Mullany LC, Khatry SK, LeClerq SC, Darmstadt GL, et al. Sex differences in morbidity and care-seeking during the neonatal period in rural southern Nepal. Journal of Health, Population and Nutrition. 2015;33(1):11.	10

63	Das MB, Amin S, Johnson K, Hossain A. Whispers to voices: Gender and social transformation in Bangladesh. 2008.	170
64	Factors that influence the implementation of e-health: a systematic review of systematic reviews (an update)	12
65	How to govern the digital transformation of health services	12
66	Healthcare professionals' competence in digitalisation: A systematic review	16
67	The Digitization of Patient Care: A Review of the Effects of Electronic Health Records on Health Care Quality and Utilization	14
68	The Digitization of Patient Care: A Review of the Effects of Electronic Health Records on Health Care Quality and Utilization	6
69	Using Health IT to Coordinate Care and Improve Quality in Safety-Net Clinics	10
70	The role of electronic medical records in improving health care quality: A quasi-experimental study	5
71	Health Outcomes and Healthcare Efficiencies Associated with the Use of Electronic Health Records in Hospital Emergency Departments: a Systematic Review	25
72	Delivering person-centered care with an electronic health record	9
73	Patient Health Record Systems Scope and Functionalities: Literature Review and Future Directions	21
74	Digital interventions to improve safety and quality of inpatient diabetes management: A systematic review	7
75	Observing Provider Utilization of Electronic Health Records to Improve Clinical Quality Metrics	21
76	Electronic health records, adoption, quality of care, legal and privacy issues and their implementation in emergency departments	37
77	Association between Electronic Medical Records and Healthcare Quality	7
78	Acceptability of Mobile Health Interventions to Increase Diabetic Risk Factor Awareness Among the Commuter Population in Johannesburg: Descriptive Cross-Sectional Study	11
79	Feasibility and effectiveness of electronic vs. paper partograph on improving birth outcomes: A prospective crossover study design	15
80	An Inventory of Digital Tools in Use by the Ministry of Health and Family Welfare in Bangladesh	21

81	Design considerations for patient-centered eHealth interventions in an underserved context: A case of health and wellbeing services within Nairobi's informal settlements in Kenya	23
82	Role of mHealth applications for improving antenatal and postnatal care in low and middle income countries: a systematic review	11
83	Feasibility of mHealth intervention to improve uptake of antenatal and postnatal care services in peri-urban areas of Karachi: a qualitative exploratory study	11
84	Effective non-drug interventions for improving outcomes and quality of maternal health care in sub-Saharan Africa: a systematic review	18
85	What Is Human Centred Design?	17
86	Software Testing Techniques: A Literature Review	6
87	The System Usability Scale: Past, Present, and Future	24
88	A review of technology acceptance and adoption models and theories	8
89	Investigating acceptance of telemedicine services through an extended technology acceptance model (TAM)	10
90	Technology acceptance model: a literature review from 1986 to 2013	12
91	Technology Acceptance Model in M-learning context: A systematic review	16
92	Acceptability of healthcare interventions: an overview of reviews and development of a theoretical framework	13
93	Applying User-Centered Design in the Electronic Health Record (EHR) to Facilitate Patient-Centered Care in Oncology	17
94	Testing Scientific Software: A Systematic Literature Review	6
95	Measuring AT Usability with the Modified System Usability Scale (SUS)	23
96	A Systematic Review of the Technology Acceptance Model in Health Informatics	8
97	Investigating acceptance of telemedicine services through an extended technology acceptance model (TAM)	10
98	Nurses’ perceptions, acceptance, and use of a novel in-room pediatric ICU technology: testing an expanded technology acceptance model	15
99	The Usage Behavior and Intention Stability of Nurses: An Empirical Study of a Nursing Information System	33
100	Acceptability of healthcare interventions: an overview of reviews and development of a theoretical framework	13

Table ST2: List of key informants

SL	Designation	Organisation
1	Programme Manager, Maternal Health	Directorate General of Health Services (DGHS)
2	Programme Manager, Newborn Health, National Newborn Health Programme and IMCI	Directorate General of Health Services (DGHS)
3	Programme Manager, Newborn Health, National Newborn Health Programme and IMCI	Directorate General of Health Services (DGHS)
4	Deputy Programme Manager, Training and Child Injury,	Directorate General of Health Services (DGHS)
5	Deputy Programme Manager, Emergency Obstetric Care Service	Directorate General of Health Services (DGHS)
6	Senior Director and Senior Scientist, Maternal and Child Health Division	icddr,b
7	International Scientist, Maternal and Child Health Division	Icddr,b

Table ST3: List of acronyms for the Power matrix mapping figure

Si no	Type	Acronym	Stakeholder	Power	Interest
1	National	DGHS-MNC&AH	Directorate General of Health Services-Maternal Newborn Child and Adolescent Health	10	9
2	National	DGHS - HSM	Directorate General of Health Services-Hospital Service Management	10	6
3	National	DGHS-MCRAH	Directorate General of Health Services-Maternal, Child, Reproductive and Adolescent Health	9	5
4	National	UNICEF	United Nations International Children's Emergency Fund	8	6
5	National	icddr,b	International Centre for Diarrhoeal Disease Research	7	10
6	National	SCI	Save the Children	7	7
7	National	UNFPA	United Nations Population Fund	7	6
8	National	OGSB	Obstetrical and Gynaecological Society of Bangladesh	6	6
9	National	WHO	World Health Organization	6	3
10	National	Ipas	Ipas Bangladesh	5	6
11	National	BMS	Bangladesh Midwifery Society	3	5
12	District	CS	Civil Surgeon	9	10
13	District	DDFP	Deputy Director Family Planning	6	2
14	District	UH&FPO	Directorate General of Health Services - Upazila Health and Family Planning Officer	8	9
15	District	UFPO	Upazila Family Planning Officer	5	2
16	District	RMO	Residential Medical Officer	7	8
17	District	Doctor	Directorate General of Health Services	7	4
18	District	Nurse	Directorate General of Health Services	7	8
19	District	Midwife	Directorate General of Health Services	5	9
20	District	SCI	Save the Children	5	7
21	District	BRAC	Bangladesh Rural Advancement Committee- Local Non-Government Organisation	3	6
22	District	Pollisree	Local Non-Government Organisation	3	5
23	District	DESA	Local Non-Government Organisation	2	4

Table ST4: List of maternal health resource documents

SL	Name of Documents	Pages
1	WHO recommendation for the prevention and treatment of postpartum haemorrhage (PPH)	48
2	Ecalmpsia and PPH Action Plan in Bangladesh	42
3	Guideline on Antenatal Care (ANC) (Bangladesh)	41
4	Maternal Health Standard Operating Procedure (SOP) Volume 1	226
5	Maternal Health Standard Operating Procedure (SOP) Volume 2	384
6	Bangladesh National Strategy for Maternal Health (English)	90
7	Bangladesh National Strategy for Maternal Health (Bangla)	90
8	Standard Clinical Management Protocols and Flowcharts on Emergency Obstetric and Neonatal Care 2019	132
9	Manual on labour room management protocol	161
10	Guideline on Intrapartum care (IPC) and Postnatal Care (PNC)	66
